# Development of Real-Time Molecular Assays for the Detection of Wesselsbron Virus in Africa

**DOI:** 10.3390/microorganisms10030550

**Published:** 2022-03-03

**Authors:** Martin Faye, Thiané Seye, Pranav Patel, Cheikh Tidiane Diagne, Moussa Moise Diagne, Moussa Dia, Fatou Diène Thiaw, Amadou Alpha Sall, Ousmane Faye

**Affiliations:** 1 Virology Department, Institut Pasteur de Dakar, 36, Avenue Pasteur, Dakar 220, Senegal; thiane.seye94@gmail.com (T.S.); CheikhTidiane.DIAGNE@pasteur.sn (C.T.D.); MoussaMoise.DIAGNE@pasteur.sn (M.M.D.); Moussa.DIA@pasteur.sn (M.D.); fatoulayethiaw25@gmail.com (F.D.T.); Amadou.SALL@pasteur.sn (A.A.S.); Ousmane.FAYE@pasteur.sn (O.F.); 2 Expert Molecular Diagnostics, 82256 Fürstenfeldbruck, Germany; patelp@gmx.de

**Keywords:** Wesselsbron virus, molecular assays, diagnostics, point-of-need, Africa

## Abstract

Wesselsbron is a neglected, mosquito-borne zoonotic disease endemic to Africa. The virus is mainly transmitted by the mosquitoes of the *Aedes* genus and primarily affects domestic livestock species with teratogenic effects but can jump to humans. Although no major outbreak or fatal case in humans has been reported as yet worldwide, a total of 31 acute human cases of Wesselsbron infection have been previously described since its first isolation in 1955. However, most of these cases were reported from Sub-Saharan Africa where resources are limited and a lack of diagnostic means exists. We describe here two molecular diagnostic tools suitable for Wesselsbron virus detection. The newly established reverse transcription-quantitative polymerase chain reaction and reverse-transcription-recombinase polymerase amplification assays are highly specific and repeatable, and exhibit good agreement with the reference assay on the samples tested. The validation on clinical and veterinary samples shows that they can be accurately used for Wesselsbron virus detection in public health activities and the veterinary field. Considering the increasing extension of *Aedes* species worldwide, these new assays could be useful not only in laboratory studies for Wesselsbron virus, but also in routine surveillance activities for zoonotic arboviruses and could be applied in well-equipped central laboratories or in remote areas in Africa, regarding the reverse-transcription-recombinase polymerase amplification assay.

## 1. Introduction

Wesselsbron virus (WSLV) is a mosquito-borne flavivirus first isolated in 1955 from the blood of a febrile man and from a dead lamb during an outbreak in the South African town of Wesselsbron [1,2]. WSLV is responsible for the Wesselsbron disease (WSL), a neglected disease associated with teratogenic effects in lambs, and abortion and mortality in pregnant ewes as the Rift Valley Fever virus. However, only a less severe fever was reported in adult WSLV-positive goats, cattle and pigs [3,4]. In addition, WSLV has also been associated with neurological damage in horses in South Africa [5] and, more recently, the virus has been isolated from rodent’s brain tissues in Senegal [6]. However, human cases were commonly characterized by a short period of fever, arthralgia and myalgia [6,7].

No major outbreak with fatal cases in humans has been reported; yet, several isolations of WSLV from mosquito populations [8,9,10,11], livestock [2,12], wildlife [13] and 31 humans [1,6,14] and serological evidence of its circulation in various hosts [15,16,17,18] were described in Sub-Saharan countries [7] and Thailand [19].

WSLV exhibited two major clades circulating in Sub-Saharan Africa [6,14] and several methods have been previously described for identification of WSLV infection including direct virus isolation in newborn mice [6], cell culture coupled with immunofluorescence [20,21] and molecular methods [6,14,22,23,24,25]. However, these methods are associated with a high workload, are time consuming and are sometimes not specific for WSLV detection [22,23,24,25].

Thus, development of reliable and specific assays is needed for detection and surveillance of WSLV not only in countries where the virus circulation has been previously reported [7,19], but also in geographical regions where it may emerge due to the presence of appropriate mosquito vectors [8,9,10,11].

Herein, sensitive fluorescent probe-based reverse transcription-quantitative polymerase chain reaction (RT-qPCR) and reverse-transcription-recombinase polymerase amplification (RT-RPA) assays have been developed and evaluated for rapid and specific detection of WSLV. The new methods were further evaluated with clinical and veterinary samples and are suitable for use in routine laboratory diagnosis of WSLV infection in both humans and animals and for field diagnosis in limited-resource settings during entomological or veterinary surveillance.

## 2. Materials and Methods

### 2.1. Ethical Statement

Mosquito pools used in this study were collected in the frame of the national integrated surveillance program for arbovirus in Senegal while archived clinical specimens were provided by the WHO Collaborating Centre for Arboviruses and Hemorrhagic Fevers in Institute Pasteur in Dakar, accredited for routine diagnostic, surveillance and animal research, according to IACUC anima [26]. The Senegalese national ethical committee approved the protocol as a less than minimal risk research, and written consent forms were not required. All viral isolations in suckling mice were performed in accordance with the ARRIVE guidelines [27].

### 2.2. Virus Stocks and Samples Collection

A selection of twenty-eight WSLV stocks previously prepared by inoculating *Aedes albopictus* continuous cell lines (C6-36) for 4 days, followed by a specific immunofluorescence assay (IFA) as previously described [28], were obtained from the collection of the WHO collaborating centre for arboviruses and viral hemorrhagic fevers (CRORA) at the Institute Pasteur of Dakar (IPD) in Senegal. Filtrated virus stocks were passed one time by intracerebral inoculation of newborn Swiss suckling mice (1–2 days old) in the animal laboratory at the IPD. Brain tissues from febrile mice were homogenized in L-15 medium (Gibco BRL, Grand Island, NY, USA) and tested by RT-qPCR as previously described [29]. In addition, eleven additional virus stocks representing seven other flaviviruses important in the public health context in Africa were also used for specificity assessment of the new assays (Table A1). In addition, a total of 50 pan-flavivirus negative mosquito pools and 20 pan-flavivirus negative human sera, collected in the field in Senegal from January to December 2019, were used for assessment of the diagnostic specificity. WSLV positive sera from two patients and brain tissues from a black rat previously collected from the field in Senegal [6] were also tested for assessment of clinical and veterinary sensitivity using both assays.

### 2.3. Primers Design

Multiple alignments of WSLV nucleotide sequences available online (www.ncbi.nlm.nih.gov/genbank/; last accessed on 11 August 2021) were carried out by using Muscle algorithm [30] within Unipro UGENE software [31]. Both primers and inverse-sense TaqMan probes were designed on the NS3 protein using Primer3web^®^ software (version 4.0.0, Whitehead Institute for Biomedical Research, Cambridge, MA, USA). According to RPA guidelines from TwistDx (Cambridge, UK), two forward primers, two reverse primers and one inverse-sense fluorescent exo probe were designed manually based on the conserved region of the envelope protein (E). To avoid non-specific cross-reactions with other flaviviruses, primers and probes were validated by BLAST analysis on NCBI (https://blast.ncbi.nlm.nih.gov//; last accessed on 15 August 2021) and oligonucleotides were produced by TIB Mol-Biol (Berlin, Germany). Four primer combinations were tested to select the RPA primers and probe set exhibiting the highest analytical sensitivity with 10^3^ molecules of the in vitro RNA standard.

Plasmids were generated at GenExpress (Berlin, Germany) by inserting the ligated E, NS3 and NS5 amplicons into pCRII (Life Technologies, GmbH, Darmstadt, Germany) and the in vitro RNA standard was synthetized with T7 RNA-polymerase by TIBMolBiol (Berlin, Germany) at a concentration of 10^8^ RNA molecules/reaction, according to the manufacturer’s recommendations. The combination with the highest and earliest start of the exponential amplification curve was selected and tested with 10-fold serial dilutions (from 10^3^ to 10 RNA molecules/reaction) of the in vitro RNA standard. The RT-qPCR primers were also tested using the same in vitro RNA standard. 

### 2.4. Samples Preparation and RNA Extraction

All virus stocks, negative sera and negative mosquito pools analyzed in this study derived from collection of the WHO Collaborating Centre for Arboviruses and Hemorrhagic fevers (CRORA) in Senegal at Institute Pasteur of Dakar (IPD). Extraction of viral RNA from 100 µL of virus stocks or ten-fold serial dilutions of virus-spiked L15-medium, serum and CSF sample was performed with the QIAamp viral RNA mini kit (Qiagen, Heiden, Germany) according to the manufacturer’s instructions. Viral RNA was eluted in a final volume of 60 μL and frozen at −80 °C prior to downstream applications. 

### 2.5. Real-Time RT-qPCR Conditions

Real-time RT-qPCR was performed in duplicates using the qScript One-step qRT-PCR Kit (Quanta Biosciences, Gaithersburg, MD, USA) in a final volume of 25 μL following the previously established protocol [32] and the reaction was carried out on a 7500 Fast Real-Time system cycler (Applied Biosystems, Foster City, CA, USA). 

### 2.6. RPA Assay Conditions

RT-RPA amplifications were achieved in a final volume of 50 μL by adding 0.2 µL of the SuperScript II Reverse Transcriptase enzyme (Invitrogen) to the TwistAmp exo kit (TwistDx, Cambridge, UK) and reduction of the volume of water in the master mix as previously described [33]. For each reaction, 45 μL of master mix was prepared in each tube lid and 5 μL of viral RNA was subsequently added to each lid tube. Then, the lids were closed carefully and the master mix was centrifuged into the rehydrated reaction pellet containing a dried enzyme using a mini spin centrifuge. The reaction was performed at 42 °C for 15 min in the Twista Tubescanner device (TwistDx, Cambridge, UK) connected to a computer for real-time monitoring of fluorescence as previously described [34].

### 2.7. Specificity Assessment

In order to evaluate the analytical specificity of the newly developed WSLV RT-qPCR and RT-RPA, RNA samples from 28 WSLV positive isolates and 11 isolates of other flaviviruses were tested in duplicates using both assays. Positive and negative controls containing positive RNA and nuclease-free water, respectively, were included in each run. In addition, a total of 50 pan-flavivirus negative mosquito pools and 20 pan-flavivirus negative human sera were also analyzed in duplicates using both assays for determination of the diagnostic specificity. A previously described pan-flavivirus RT-qPCR assay was used as a reference test [29]. 

### 2.8. Sensitivity Assessment

#### 2.8.1. Analytical Sensitivity

The limit of detection of the newly established Wesselsbron virus RT-qPCR and RT-RPA assays was assessed with data from the eight runs on a dilution range of the in vitro RNA standard (10^8^–1 RNA molecules/reaction). A linear regression analysis and a semi-log regression analysis were performed for the RT-qPCR assay and the RT-RPA assay, respectively. The probit regression analysis was also performed using data of eight runs from both assays to determine the limit of detection at 95% probability. The graphs were plotted using PRISM (GraphPad Software Inc., San Diego, CA, USA).

#### 2.8.2. Sensitivity in Human Serum and L-15 Medium

Ten-fold serial dilutions of two virus stocks with known titer (pfu/mL) were prepared in L-15 medium and human serum (Sigma-Aldrich, Saint-Louis, MO, USA) and analyzed in triplicates using both assays to determine diagnostic sensitivity of the newly developed assays. Regression curves were obtained representing the pfu/reaction versus the threshold cycle (Ct) and threshold time (Tt) values for the RT-qPCR and the RT-RPA, respectively. The lowest titer with amplification was considered as the analytical limit of detection (LOD). The amplification efficiency was calculated for both assays from the slope of the pfu/reaction regression lines (E = 10^1/slope^ – 1).

In addition, extracted RNA from a virus stock with a titer was analyzed eight times in the same run and in eight different runs to determine intra-assay and inter-assay coefficients of variation (CV).

#### 2.8.3. Sensitivity on Clinical and Veterinary Samples

Extracted RNA from two WSLV positive human sera and brain tissues from one rodent isolated in Senegal were tested in duplicates for confirmation of the reliability of the newly established assays. The pan-flavivirus RT-qPCR assay was used as a reference test [29]. 

### 2.9. Assessment of the Impact of Genetic Diversity on Assays

In silico analysis of both assays was performed again sequences of WSLV isolates from South Africa and Senegal obtained from GenBank (www.ncbi.nlm.nih.gov/genbank/; last accessed on 15 August 2021), using the MAFFT alignment algorithm implemented in the Unipro UGENE software [31].

### 2.10. Statistical Analysis

The limit of detection of the WSLV assays was calculated by performing a probit regression analysis on the data set of eight RPA assays using STATISTICA software (StatSoft, Hamburg, Germany) in order to determine the number of RNA molecules/reaction at 95% of probability. The correlation between regression curves obtained from serial dilutions in L-15 medium and human serum was determined for each assay using the Pearson correlation test where a coefficient of 1 represents a good correlation. A kappa test was used to compare detection performances of the newly established assays and the pan-flavivirus RT-qPCR assay [29], where the Cohen’s kappa coefficient (k) represents a measure of the agreement between assays with a 95% confidence interval and a *p* < 0.05 is considered to be statistically significant. In addition, positive and negative predictive values (PPV and NPV), diagnostic sensitivity and specificity were calculated using standard formulas [35]. The PPVs and NPVs were compared between the RT-qPCR and the RT-RPA assays using Fisher’s exact test; considering a *p* < 0.05 as statistically significant.

## 3. Results

### 3.1. Primers Selection

In silico analysis of the primers and probes designed herein using BLAST exhibited no possible cross-reactivity with none-WSLV sequences on NCBI. The RT-qPCR primers (WSBFOW/WSBREV) detected well the dilution 10^3^ RNA molecules/reaction of the in vitro RNA standard. For the RT-RPA assay, the four combinations of the RPA primers were also screened with the 10^3^ RNA molecules/reaction of the in vitro transcribed RNA standard. The combination with the highest and earliest start of the exponential amplification curve was selected and tested with 10-fold serial dilutions (from 10^3^ to 10 RNA molecules/reaction) of the in vitro RNA standard. Fortunately, the primer pair (RF2/RR2) enabled detection down to 10^2^ RNA molecules/reaction of the in vitro RNA standard. Therefore, it was selected for further assay validation (Table 1). 

### 3.2. Analytical Specificity

All 28 WSLV positive isolates were detected using both assays while amplification was not observed for any other none-WSLV strain, resulting then in an analytical specificity of 100% for the newly established assays (Table 2). 

### 3.3. Analytical Sensitivity

**Figure 1:**
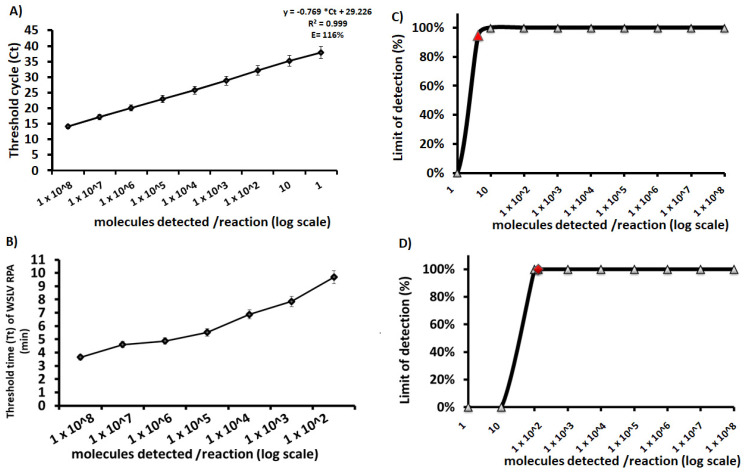
Analytical sensitivity of the newly established Wesselsbron virus RT-qPCR and RT-RPA assays. A linear regression analysis and a semi-log regression analysis were performed by plotting the RT-qPCR threshold cycle values (Ct) (**A**) and the RT-RPA time threshold (Tt) (**B**), respectively, against the number of RNA molecules per reaction detected in eight replicates (8/8). The dots represent the mean values and the error bars represent the standard deviation. The RT-qPCR assay produced positive results with dilutions 10^8^ to 10 RNA molecules/reaction on 8 out of 8 runs and detected 1 molecule/reaction on 5 out of 8 runs. However, the RT-RPA assay produced positive results until 100 molecules/reaction between 3 and 10 min. The probit regression analysis was performed using data of eight RT-qPCR assay runs (**C**) and eight RPA assay runs (**D**). The graphs were plotted using PRISM (GraphPad Software Inc., San Diego, CA, USA) and the limit of detection at 95% probability is depicted by the red triangle. The limit of detection at 95% probability is of 4 and 130 RNA molecules/reaction for the RT-qPCR and RT-RPA assays, respectively.

The analytical sensitivity of the newly established Wesselsbron virus RT-qPCR and RT-RPA assays was determined with Ct and Tt data values from eight sets of ten-fold dilutions of the in vitro RNA standard ranging from 10^8^ to 1 molecules/reaction. The WSLV RT-qPCR assay detected the in vitro RNA standard with the concentration from 10^8^ to 10 molecules/reaction in all eight RPA runs and the concentration of 1 molecule/reaction in five runs (lmtest *p* = 3.427 × 10^−13^) (Figure 1A). However, the RT-RPA assay produced positive results until dilution of 100 molecules/reaction in eight replicates, while no amplification was observed in the tube containing ten and one molecule/reaction. Ten min is the maximum time needed to amplify as low as 100 RNA molecules by the RT-RPA assay (Figure 1B). With these data sets, a probit regression analysis was performed and revealed a detection limit at 95% probability of 4 and 130 RNA molecules/reaction for the newly established RT-qPCR and RT-RPA assays, respectively (Figure 1C,D).

### 3.4. Diagnostic Performances

All the 50 mosquito pools and 20 human sera which tested negative for the pan-flavivirus RT-qPCR, also tested negative for both new WSLV assays. However, all the 31 positive samples including 28 WSLV viral stocks, 2 human sera and 1 rodent brain tissues, were detected using both diagnostic tools in a mean Tt value of 43.31 ± 0.18 min (ranging from 31.99 to 51.12), 37.74 ± 0.16 min (ranging from 31.92 to 46.73) and 4.55 ± 0.25 min (ranging from 3.21 to 5.78) for the pan-flavivirus RT-qPCR, the WSLV RT-qPCR and the WSLV RT-RPA, respectively (Table 2). Using the standard formulas [26], NPVs and PPVs of 1 and an accuracy of 100% (95% CI; 96.41–100%) were determined for both newly established RT-qPCR and RT-RPA assays (Fisher’s exact test *p* < 0.0001). In addition, a diagnostic specificity of 100% (95% CI; 94.87–100%) and a diagnostic sensitivity of 100% (95% CI; 88.78–100%) were determined for both WSLV RT-qPCR and RT-RPA assays. Both new assays also showed good agreement with the real-time pan-flavivirus RT-qPCR [26] used as reference test (Cohen’s Kappa test, k = 1 ± 0.09 (95% CI; 0.80–1.20); *p* < 0.05), indicating that the newly established WSLV assays are accurate and give 100% concordance to results obtained with the real-time pan-flavivirus RT-qPCR [29] on the same samples (*p* < 0.0001). In addition, intra-run and inter-run CV of 0.009 and 0.007, respectively, were found for the newly established RT-qPCR assay while the RT-RPA assay showed intra-run and inter-run CV of 0.002 and 0.001, respectively, indicating that these assays are highly repeatable.

### 3.5. Sensitivity in Human Serum and L-15 Medium

**Figure 2:**
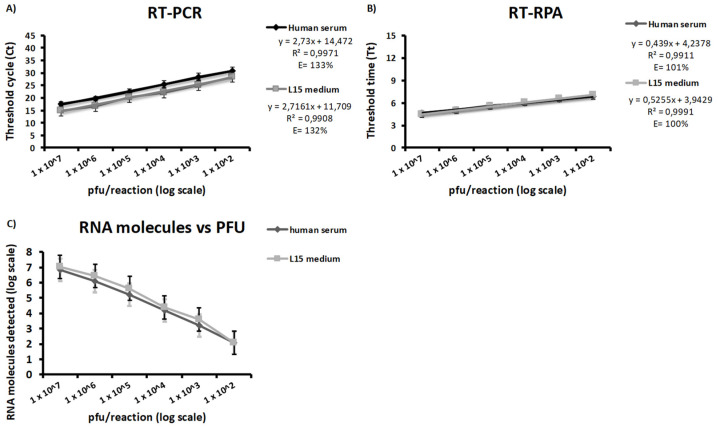
Diagnostic sensitivity of the newly established RT-qPCR (**A**) and RT-RPA (**B**) assays using serial 10-fold dilutions of Wesselsbron virus in human serum (black curve) and L-15 medium (gray curve). Dilutions were tested in triplicates using both assays. Both assays detected until 100 pfu, corresponding to 100 RNA molecules using the equation obtained in Figure 1A (**C**).

Ten-fold dilutions of two WSLV stocks prepared in human serum and L-15 medium were analyzed in triplicates using both the WSLV-specific RT-qPCR and RT-RPA assays. The new RT-qPCR assay yielded a sensitivity down to 100 pfu/reaction in both human serum and L-15 medium within a mean Tt of 47.63 ± 4.82 min (40.12–55.34) and 44.39 ± 4.57 min (37.15–52.74), respectively, while the new RT-RPA assay exhibited an LOD of 100 pfu/reaction in both human serum and L-15 medium within a mean Tt of 6.81 ± 0.12 min and 7.08 ± 0.52 min, respectively. Efficiencies of 132% and 133% were shown by the new RT-qPCR assay for dilutions in human serum and dilutions in L-15 medium, respectively, while the new RT-RPA assay exhibited efficiencies of 101% and 100%, respectively (Figure 2A,B). Pearson’s correlation coefficients of 0.9939 (*p* = 5.493 × 10^−5^) and 0.9967 (*p* = 1.606 × 10^−5^) were also determined between regression lines from dilutions in human serum and L-15 medium tested with the new RT-qPCR and RT-RPA assays, respectively. Both assays detected until 100 pfu, corresponding to 100 RNA molecules calculated from Ct values and the equation obtained from the linear regression analysis of eight RT-qPCR data sets of ten-fold dilutions of the molecular standard RNA (Figure 2C).

### 3.6. Sensitivity in Clinical and Veterinary Samples

All collected samples were screened in duplicates with both assays. As the reference pan-flavivirus RT-qPCR method, all three positive specimens tested positive with both WSLV assays. The WSLV-specific RT-qPCR detected the RNA in both human sera and rodent’s brain tissues in a mean Tt of 44.51 ± 0.51 min (43.78–45.88), while the RT-RPA assay gave amplification with these samples in a mean time of 7.46 ± 0.15 min (7.30–7.60); indicating that these assays are highly sensitive on clinical and veterinary samples and six times faster than the RT-qPCR (Table 3).

### 3.7. In Silico Analysis of New Primers and Probes Sequences

**Figure A1:**
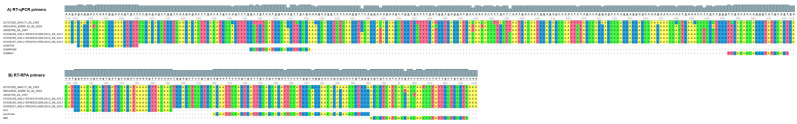
In silico analysis of (**A**) the newly established RT-qPCR assay and (**B**) the RT-RPA assay against sequences of Wesselsbron virus isolates from South Africa (SA) and Senegal (SN), respectively. The alignment was performed using the MAFFT algorithm implemented in the Unipro UGENE software [31]. The in silico analysis revealed more signature erosion in the RT-qPCR primers when compared to sequences from South Africa.

Available coding-complete sequences from South Africa (SA) and Senegal (SN) enabled in silico evaluation of the newly developed WSLV-specific assays using the BLAST program (https://blast.ncbi.nlm.nih.gov/, accessed on 1 November 2021). A total of 7 and 6 mismatches between the targeted WSLV gene sequence and the currently available sequences have been identified for the new RT-qPCR and RT-RPA assays, respectively. The forward and reverse primers of the RT-qPCR assay (WSBFOW and WSBREV) are highly similar to the target region of the sequences from Senegal, while they show a dissimilarity of 0% and 5% against the isolates from South Africa. The RT-qPCR assay’s probe (WSBPROBE) reveals a dissimilarity of 5% to sequences from Senegal, while it is more distant to the isolate EU707555_SAH177_SA_1955 from South Africa with a dissimilarity of 15% (Table A2; Figure A1). The forward and reverse primers of the RT-RPA assay (RF2 and RR2) were also highly similar to the sequences from Senegal, while the reverse primer reveals dissimilarities of 3%, 3% and 6% to the isolates from South Africa. The probe of the RT-RPA assay (exoProbe) shows a dissimilarity of 2% to the Senegalese sequences. However, it is distant from the isolates from South Africa with dissimilarities of 2%, 2% and 8% (Table A2; Figure A1). Nevertheless, these dissimilarities did not omit detection of the aligned sequences from Senegal by the new WSLV assays (Table 3). 

## 4. Discussion

WSL is a neglected, mosquito-borne infection reported in Africa [6,14] with symptoms close to those caused by Rift Valley Fever infection in animals [14]. In addition, the geographic distribution of WSL remains sparsely determined [6,7,14,19]. Rare data are currently available from Africa [13] where resources, infrastructure and diagnostic capacities are limited. In addition, the clinical diagnosis of WSLV infection is difficult as it is at least always confused with the clinical presentation of Rift Valley Fever disease. Therefore, we developed two real-time molecular assays for rapid detection of WSLV. These assays are based on conserved regions on NS3 and E Wesselsbron virus proteins.

The newly established assays are highly specific for WSLV detection and showed good agreement with the pan-flavivirus RT-qPCR [29]. In addition, our WSLV assays showed excellent diagnostic performances, which indicates that they are highly sensitive, similar to the pan-flavivirus RT-qPCR [29]. The newly established RT-RPA assay is much faster than the pan-flavivirus RT-qPCR [29] and needs less than 6 min for WSLV detection in all isolates that tested positive. Both protocols require extraction and thus actual time saved could be a lot less significant. However, the RT-RPA assay could be used with rapid extraction methods which had previously shown best performances in extraction of viral RNA [36] and were applied as the first-line assay in low-resource settings.

The recorded inter-assay and intra-assay variability below 1% also ensures high repeatability for the two new assays. A total of 7 and 6 mismatches between the targeted WSLV gene sequence and the currently available sequences were identified for the new RT-qPCR and RT-RPA assays, respectively. However these mismatches are mostly bases belonging to the same nucleotide category and are not present at positions critical for binding of the oligonucleotides [37]. However, the performances of the new WSLV-specific assays could be further assessed on isolates from South Africa. 

Our WSLV RT-qPCR assay could be applied in well-equipped central laboratories for routine WSLV diagnosis as complementary to an existing method [38] while the RT-RPA assay showed more advantages in remote areas where highly equipped laboratories are not available, since it uses cold-chain independent reagents and is stable under different environmental conditions including temperatures above 30 °C [39]. However, our RT-RPA assay is about 50 times less sensitive than the RT-qPCR. Thus, in order to prevent risks of misdiagnosis for patients, the RT-qPCR assay could also be applied as a confirmation method on samples that were tested negative by RT-RPA assay. Our WSLV-specific assays appear then as appropriate tools for the point-of-need detection of acute WSLV cases as they are highly sensitive and cover the currently known diversity of WSLV in West Africa [6].

Although no fatal human case has been identified yet, these new tools could be used not only in a context of other investigations in areas where the virus circulation has been previously reported [7,19], but also in active entomological, veterinary and clinical surveillance studies ruled out in regions where the *Aedes* vectors have been previously identified [8,9,10,11,40], in order to determine its viral pathogenesis, identify its potential reservoirs and estimate the real public and veterinary health impact of this disease, at least in Africa. 

## Figures and Tables

**Table 1:** Description of the newly developed primers and probes.

Name	Sequence 5′-3′	Protein	Position ^a^	GC%	Tm ^b^	Product Size
WSBFOW	GAGGACCAACGGAAAGTGTT	NS3	6146–6165	50.00	59.04	231
WSBREV ^c^	ACTGCATACCCTGGTGTCAA	NS3	6357–6376	50.00	59.01	
WSBPROBE ^c^	6FAM-TCGCAACCTGCCATGACAGC--BBQ	NS3	6202–6221	60.00	68.05	
RF2	GGAACAGCAGTGATGCAGTAAAAGTTACAAC	Envelope	1895–1926	50.00		126
RR2 ^c^	GACGCAGCAATAGGGTTGGTCGTGATGAGCT	Envelope	1991–2022	50.00		
exoProbe ^c^	CCCACGGTTCTCTGTTCCTGCCATGGAG dT-BHQ1-THF–dT-FAM GCTGCAATCACTGGA-Ph	Envelope	1940–1990	60.00		

FAM, fluorescein amidite; BBQ, blackberry quencher; BHQ1, black hole quencher 1; Ph, phosphate; THF, tetrahydrofuran; ^a^, corresponding nucleotide positions of WSLV strain SAH177 (GenBank Ac. No. EU707555); ^b^, melting temperature; ^c^, sequence in reverse sense.

**Table 2:** Analytical specificity of the newly developed RT-qPCR and RT-RPA assays.

Strains	Virus	PAN-FLAVIVIRUS RT-qPCR		WSLV RT-qPCR		WSLV RT-RPA	
		Mean Ct Value ^a^	SD	Mean Ct Value ^a^	SD	Mean Tt Value ^b^	SD
ArD142157	Wesselsbron	25.80	0.01	26.18	0.58	4.21	0.21
ArD142585	Wesselsbron	21.81	0.02	23.79	0.29	4.99	0.11
ArD140166	Wesselsbron	26.07	0.24	24.81	0.03	3.30	0.11
ArD142730	Wesselsbron	29.11	0.18	27.52	0.30	4.43	0.40
ArD142098	Wesselsbron	22.93	0.63	23.12	0.27	4.83	0.04
ARA23495	Wesselsbron	28.49	0.48	19.36	0.15	5.33	0.02
ArD90431	Wesselsbron	24.33	0.24	15.18	0.01	3.56	0.47
ArD90535	Wesselsbron	25.06	0.23	15.10	0.07	3.21	0.42
ArD142143	Wesselsbron	31.41	0.26	26.36	0.38	3.65	0.02
ArD65233	Wesselsbron	23.69	0.06	14.93	0.04	3.54	0.53
ArD142775	Wesselsbron	24.67	0.22	22.77	0.44	3.21	0.06
ArD140179	Wesselsbron	28.79	0.06	15.04	0.01	3.37	0.00
ArD92269	Wesselsbron	25.54	0.07	15.89	0.13	4.94	0.23
ArD142716	Wesselsbron	24.41	0.01	24.77	0.04	4.83	0.32
ArD140184	Wesselsbron	21.49	0.03	14.71	0.01	4.15	0.76
ArA20858	Wesselsbron	29.98	0.30	24.57	0.01	5.49	0.38
SH88963	Wesselsbron	24.31	0.15	19.75	0.13	4.84	0.09
ArA22079B	Wesselsbron	22.69	0.04	24.67	0.20	5.49	0.64
ArD141023	Wesselsbron	22.34	0.18	25.79	0.43	5.42	0.02
ArD285495	Wesselsbron	14.78	0.14	27.59	0.13	5.78	0.06
ArD90416	Wesselsbron	24.89	0.23	15.26	0.01	5.65	0.47
ArD140187	Wesselsbron	27.77	0.05	15.45	0.01	5.39	0.02
ArD90541	Wesselsbron	24.13	0.20	15.37	0.02	5.50	0.57
ArD92276	Wesselsbron	25.58	0.04	15.72	0.01	4.94	0.06
ArD140162	Wesselsbron	23.43	0.23	15.52	0.07	4.21	0.56
ArD140194	Wesselsbron	21.68	0.04	14.71	0.04	4.83	0.08
ArD85094	Wesselsbron	21.96	0.11	14.92	0.01	4.85	0.19
ArA16523	Wesselsbron	22.29	0.05	14.80	0.02	3.44	0.08
ArA 23139	Bagaza	26.11	0.02	−	–	–	–
ArD76986	WNV Lineage 1	23.09	0.01	–	–	–	–
B956	WNV Lineage 2	16.59	0.01	–	–	–	–
ArD96655	WNV Koutango	23.25	0.01	–	–	–	–
ArD94343	WNV new Lineage	19.39	0.05	–	–	–	–
New Guinea C	DENV2	22.98	0.00	–	–	–	–
H-241	DENV4	25.65	0.01	–	–	–	–
ArAAMT/7	Yellow fever	24.80	0.03	–	–	–	–
MR766	Zika	27.88	0.01	–	–	–	–
SAAR1776	Usutu	28.59	0.04	–	–	–	–
ArB1803	Usutu subtype	19.83	0.02	–	–	–	–

SD: standard deviation; Ct or Tt: threshold cycle or time; ^a^: mean Ct value obtained with duplicates; ^b^: mean Tt value (minutes) obtained with duplicates. WNV: West Nile virus. DENV: dengue virus.

**Table 3:** Sensitivity of the newly developed RT-qPCR and RT-RPA assays on clinical and veterinary samples.

Strains	GenBank Accession Number	Species	Sample Type	WSLV RT-qPCR			WSLV RT-RPA	
				Mean Ct Value ^a^	Mean Tt Value ^b^	SD	Mean Tt Value ^b^	SD
WSLV-IP262451/SEN/2014	KY056257	human	Serum	26.18	43.78	0.02	7.30	0.70
WSLV-IP248525/SEN/2013	KY056256	human	Serum	23.79	45.88	015	7.60	0.18
WSLV-IP259570/SEN/2013	KY056258	rodent	Brain tissues	24.81	43.87	1.37	7.50	0.15

SD: standard deviation. Ct or Tt: threshold cycle or time; ^a^: mean Ct value obtained with duplicates; ^b^: mean Tt value (minutes) obtained with duplicates.

## Data Availability

All the data supporting reported results can be found in the manuscript.

## Appendix A

**Table A1:** Description of virus stocks used in our study.

Strains	Virus	Place of Collection	Year of Collection	Species	Reference
ArD142157	Wesselsbron	Senegal	1995	*Aedes dalzieli*	CRORA database
ArD142585	Wesselsbron	Senegal	1995	*Aedes dalzieli*	CRORA database
ArD140166	Wesselsbron	Mauritania	1995	*Aedes vexans*	CRORA database
ArD142730	Wesselsbron	Senegal	1995	*Aedes dalzieli*	CRORA database
ArD142098	Wesselsbron	Senegal	1995	*Aedes dalzieli*	CRORA database
ARA23495	Wesselsbron	Côte d’Ivoire	1987	*Culex perfuscus*	CRORA database
ArD90431	Wesselsbron	Senegal	1988	*Aedes vittatus*	CRORA database
ArD90535	Wesselsbron	Senegal	1988	*Aedes minutus*	CRORA database
ArD142143	Wesselsbron	Senegal	1995	*Aedes dalzieli*	CRORA database
ArD65233	Wesselsbron	Senegal	1995	*Aedes dalzieli*	CRORA database
ArD142775	Wesselsbron	Senegal	1995	*Aedes dalzieli*	CRORA database
ArD140179	Wesselsbron	Mauritania	1995	*Aedes vexans*	CRORA database
ArD92269	Wesselsbron	Senegal	1988	*Cellia domicola*	CRORA database
ArD142716	Wesselsbron	Senegal	1995	*Aedes dalzieli*	CRORA database
ArD140184	Wesselsbron	Mauritania	1995	*Aedes vexans*	CRORA database
ArA20858	Wesselsbron	Côte d’Ivoire	1982	*Culex perfuscus*	CRORA database
SH88963	Wesselsbron	Senegal	1988	*Human*	CRORA database
ArA22079B	Wesselsbron	Côte d’Ivoire	1984	*Stegomyia africanus*	CRORA database
ArD141023	Wesselsbron	Senegal	1995	*Aedes dalzieli*	CRORA database
ArD285495	Wesselsbron	Senegal	2016	*Culex perfuscus*	CRORA database
ArD90416	Wesselsbron	Senegal	1988	*Stegomyia luteocephalus*	CRORA database
ArD140187	Wesselsbron	Mauritania	1995	*Aedes vexans*	CRORA database
ArD90541	Wesselsbron	Senegal	1988	*Aedes minutus*	CRORA database
ArD92276	Wesselsbron	Senegal	1988	*Cellia pharoensis*	CRORA database
ArD140162	Wesselsbron	Mauritania	1995	*Aedes vexans*	CRORA database
ArD140194	Wesselsbron	Mauritania	1995	*Aedes vexans*	CRORA database
ArD85094	Wesselsbron	Senegal	1987	*Aedes dalzieli*	CRORA database
ArA16523	Wesselsbron	Côte d’Ivoire	1985	*Culex perfuscus*	CRORA database
ArA 23139	Bagaza	Côte d’Ivoire	1988	*Culex poicilipes*	CRORA database
ArD76986	WNV Lineage 1	Senegal	1990	*Culex poicilipes*	KJ131500
B956	WNV Lineage 2	Uganda	1937	*Human*	AY532665
ArD96655	WNV Koutango	Senegal	1993	*Rhipicephalus guihoni*	KJ131501
ArD94343	WNV new Lineage	Senegal	1992	*Culex perfuscus*	KJ131502
New Guinea C	DENV2	New Guinea	1974	*Human*	AF038403
H-241	DENV4	Philippines	1956	*Human*	U18433
ArAAMT/7	Yellow fever	Côte d’Ivoire	1973	*Aedes africanus*	CRORA database
MR766	Zika	Uganda	1947	*Rhesus Monkey*	AY632535
SAAR1776	Usutu	South Africa	1959	*Culex neavei*	AY453412
ArB1803	Usutu subtype	CAR	1969	*Culex perfuscus*	KC754958

**Table A2:** Dissimilarities between primers and currently available Wesselsbron virus sequences.

Assay	Primers	EU707555_ SAH177_SA_1955	MK163943_SA999-10_SA_2010	JN226796_SA_1997	KY056258_ WSLV-IP259570/SEN/2013_SN_2013	KY056256_ WSLV-IP248525/SEN/2013_SN_2013	KY056257_ WSLV-IP262451/SEN/2014_SN_2014
RT-qPCR	WSBFOW	5%	5%	0%	0%	0%	0%
	WSBPROBE	15%	0%	5%	5%	5%	5%
	WSBREV	5%	0%	0%	0%	0%	0%
RT-RPA	RF2	0%	0%	0%	0%	0%	0%
	exoProbe	8%	2%	2%	2%	2%	2%
	RR2	6%	3%	3%	0%	0%	0%
